# Patients with inflammatory bowel disease are more hesitant about Coronavirus disease 2019 vaccination

**DOI:** 10.3389/fmed.2022.1005121

**Published:** 2022-11-15

**Authors:** Hyuk Joon Kwon, Katherine Panagos, Madeline Alizadeh, Mack Bell, Mohammad Bourmaf, Erin Zisman, Pinkle Paul, Lauren Sibel, Uni Wong

**Affiliations:** ^1^Department of Internal Medicine, University of Maryland Medical Center, Baltimore, MD, United States; ^2^Institute for Genome Sciences, University of Maryland School of Medicine, Baltimore, MD, United States; ^3^Department of Gastroenterology and Hepatology, University of Maryland School of Medicine, Baltimore, MD, United States; ^4^Department of Gastroenterology and Hepatology, Veterans Affairs Maryland Health Care System, Baltimore, MD, United States

**Keywords:** vaccine hesitancy, immunosuppression, COVID-19, inflammatory bowel disease, vaccines

## Abstract

Despite the impact of the Coronavirus Disease 2019 (COVID-19) pandemic, vaccine hesitancy remains common in the general public and patients with Inflammatory Bowel Diseases (IBD). We sought to examine the reasons for vaccine hesitancy in patients with IBD. In this case-control study, we performed a retrospective chart review of 1,349 IBD patients and 215 non-IBD patients seen at University of Maryland Medical Center, a tertiary referral medical center, between March 2020 and October 2021. Data obtained included demographics, vaccination records, disease history, number of IBD-related surgeries, and IBD medications. 813/1,349 (60.3%) IBD patients received at least one dose of either the Pfizer/BioNTech, Moderna, or Johnson & Johnson vaccines. In a multivariate logistic regression, COVID vaccination was found to be positively associated with older age (*p-*value = 1.65e-5), female sex (*p* = 0.00194), Asian and White races (*p* = 0.02330, 0.00169), number of clinic visits (*p* = 1.11e-08), and biologic use (*p* = 7.82e-5). There was no association between vaccination and other types of vaccination nor with the use of other IBD medications. There was a negative association between vaccination status and the total number of IBD related surgeries (*p* = 0.02857). In non-IBD patients, only the number of clinic visits was positively associated with COVID-19 vaccination. Although the majority of IBD patients are immunosuppressed, COVID-19 vaccination rate was only 60.3%. Younger adults, males, African Americans, and those requiring IBD-related surgeries were less likely to receive COVID-19 vaccine. Healthcare providers need to recognize these potential risk factors for COVID-19 vaccine hesitancy.

## Introduction

When Coronavirus disease 2019 (COVID-19) vaccines became available, adults who were on immunosuppressive medications were among the earlier groups recommended by the Centers for Disease Control and Prevention to receive the vaccines. Among these groups were patients diagnosed with Inflammatory Bowel Diseases (IBD), which includes Crohn's Disease and Ulcerative Colitis. These are diseases of innate and adaptive immune system dysregulation leading to chronic intestinal inflammation. An estimated 3.1 million adults (1) in the United States live with IBD and many patients require immunosuppressive medications such as corticosteroids, immunomodulators, anti-Tumor Necrosis Factors, and other biologic agents. These medications have been associated with increased susceptibility to infections (2–5). Consequently, the fear of COVID-19 infection in patients with IBD is more pronounced, especially in those taking immunosuppressants (6). Despite the global impact of the COVID-19 pandemic, vaccine hesitancy remains in both the general public (7, 8) and the IBD population (9).

Vaccine hesitancy in IBD patients is not a novel concern to gastroenterologists. Prior studies have shown that IBD patients are less likely to receive vaccinations made necessary from their immunocompromised state (10). Some concerns from the past have transferred to COVID-19 vaccines. Studies in the United States (U.S.) and Europe reported COVID-19 vaccination intent among IBD patients as rates ranging from 54 to 96.4% (9, 11–14). Many IBD patients voiced concerns that the nature of their disease may trigger worse adverse side-effects from the COVID-19 vaccine and/or the vaccine will cause an IBD flare. Others were concerned about the overall efficacy and validity of the vaccines (15, 16). However, consensus among physicians is in support (17) of vaccination of all IBD patients as data shows COVID-19 vaccines available in the U.S. are safe (18–21) and effective (22–25) for these patients.

Recent studies have shown that the rate of COVID-19 infection in IBD patients is similar to the rate of infection in the general population (26–28). They have also shown that risk factors for adverse COVID-19 outcomes in IBD patients are also similar to the risk factors of the general population which include age and comorbidities (29–33). There have been varying reports of the effects of IBD therapies on COVID-19 infection outcomes. Some studies have found that IBD medications are not associated with more adverse COVID-19 infections (34). However, several other studies have shown 5-ASA/Sulfasalazine (31, 35) systemic corticosteroids (31, 32, 35, 36) and thiopurines (35) may be associated with worse clinical outcomes. Interestingly, anti-TNF drugs and biological therapies have not been shown to be associated with worse clinical outcomes (32, 34, 36).

Therefore, given the vulnerable nature of IBD patients in the setting of the ongoing COVID-19 pandemic, it is important to address any vaccine hesitancy seen in patients with inflammatory bowel disease and use this information to then increase rates of vaccination. We sought to investigate the barriers to vaccination by examining rate of vaccine hesitancy in patients with IBD as well as associated demographic and socioeconomic risk factors.

## Materials and methods

This study was a single-center, retrospective analysis of 1,349 patients with IBD who were seen at the University of Maryland Medical Center between March 2020 and October 2021. 215 non-IBD patients, also seen in the same clinic, were used as the control group. The period between January 2020 and October 2021 was selected to account for the disruption of the pandemic on patients' clinic appointments. This period maximizes patient capture and ensures inclusion of those who presented to clinics infrequently; patients on therapies like mesalamine are seen yearly. Furthermore, as this was a retrospective chart review on an electronic medical record system, we were able to obtain current vaccine records even in patients only seen prior to when vaccines were first made available in January 2021. The diverse control group encompasses every patient seen at the clinic without IBD. They range from patients presenting for common and general gastroenterological needs to those with cancer and/or end stage diseases. Their medication use also vary, and can range from no medications to immunosuppressants/chemotherapy.

Data was obtained by performing a chart review of the electronic health record, *Epic*^®^. The data that was collected included demographics (age, sex, race, marital status, employment, insurance type), substance use (tobacco, alcohol, illicit drugs), IBD diagnosis, year of diagnosis, number of years since diagnosis, number of IBD-related surgeries, and IBD therapy received between October 2020 and 2021 including biologics, steroids, mesalamine, thiopurines or methotrexate. The number of IBD clinic visits and IBD-related gastroenterology procedures between October 2020 and 2021 were also reported (Table 1). Information was obtained on prior COVID-19 infection and recommended vaccines including the influenza vaccine, 23-valent pneumococcal polysaccharide vaccine, 13-valent pneumococcal conjugate vaccine, recombinant zoster vaccine, and COVID-19 vaccines. Patients were recorded as having received a COVID-19 vaccine if they received at least one dose (the first dose of the two-dose series for the Pfizer/BioNTech or Moderna COVID-19 vaccines, or the single dose Johnson & Johnson COVID-19 vaccine) (Table 2).

**Table 1:** Patient demographics including IBD history.

	**IBD patients**	**Non-IBD patients**	***P*-value**
	Total (*n* = 1349)	Total (n = 215)	
**Age, mean (IQR)**	43.9 (31–55.5)	53.35 (43–64.5)	3.083e-13
**Female**, ***n*** **(%)**	711 (52.7)	141 (65.6)	0.0005077
**Race**, ***n*** **(%)**			3.445e-10
**White**	1,031 (76.4)	118 (54.9)	
**Black**	252 (18.7)	82 (38.1)	
**Hispanic**	35 (2.6)	8 (3.7)	
**Asian**	29 (2.1)	6 (2.8)	
**American Indian/Alaska Native**	1 (0.0007)	0	
**Unknown**	1 (0.0007)	1 (0.47)	
**Marital Status**, ***n*** **(%)**			0.8008
**Single**	685 (50.8)	112 (52)	
**Married**	663 (48.9)	103 (47.9)	
**History Of Substance Use**, ***n*** **(%)**			
**Alcohol Abuse**			0.3933
**Never**	1,248 (92.5)	203 (94.4)	
**Current**	74 (5.5)	7 (3.3)	
**Former**	27 (2.0)	5 (2.3)	
**Tobacco Abuse**			0.4328
**Never**	873 (64.7)	130 (60.4)	
**Current**	344 (25.5)	60 (27.9)	
**Former**	132 (9.8)	25 (11.6)	
**Illicit Drug Abuse**			0.5424
**Never**	1,123 (83.2)	173 (80.5)	
**Current**	158 (11.7)	30 (14.0)	
**Former**	68 (5.0)	12 (5.6)	
**Employment Status**, ***n*** **(%)**			0.0002292
**Employed**	870 (64.5)	109 (50.7)	
**Insurance**, ***n*** **(%)**			2.547e-05
**Commercial**	1,011 (74.9)	147 (68.4)	
**Medicaid**	139 (10.3)	11 (5.1)	
**Medicare**	191 (14.1)	57 (26.5)	
**None**	8 (0.006)	0 (0)	
**IBD phenotype**, ***n*** **(%)**
**Crohn's Disease**	908 (67.3)		
**Ulcerative Colitis**	391 (29.0)		
**Indeterminant Colitis**	50 (3.7)		
**Years Since Diagnosis, mean (IQR)**	14.71 (6–21)		
**IBD therapy**, ***n*** **(%)**
**Biologics**	992 (73.5)	4 (1.9)	2.2e-16
**Steroids**	232 (17.2)	24 (11.2)	0.0239
**Immunomodulators**	224 (16.6)	7 (3.3)	3.904e-09
**5-Aminosalicylates**	233 (17.3)	3 (1.4)	4.813e-13
**Clinic visits in 1 year, mean (IQR)**	1.82 (IQR 1-2)	1.5 (IQR 1-2)	0.00196
**IBD-related surgeries, mean**	0.8218		
**Endoscopic procedures, mean**	0.551	0.619	0.328

IQR, Interquartile Range.

**Table 2:** Vaccination and COVID history.

**Vaccination, *n* (%)**	**IBD Patients**	**Non-IBD Patients**	***P-*value**
**COVID-19**	813 (60.3)	145 (67.4)	0.2154
**Influenza**	1,170 (86.7)	170 (79.1)	0.01043
**PCV13/ PPV23**			2.2e-16
**Yes**	929 (68.9)	76 (35.3)	
**No**	381 (28.2)	120 (55.8)	
**Not Indicated**	14 (1.0)	87 (40.5)	
**Unknown**	25 (1.9)	9 (4.2)	
**Shingles**			4.283e-14
**Yes**	248 (18.4)	42 (19.5)	
**No**	793 (58.8)	76 (35.3)	
**Not Indicated**	303 (22.5)	10 (4.7)	
**Unknown**	5 (0.4)	9 (4.2)	
**Prior COVID-19 Infection**, ***n*** **(%)**	98 (7.3)	24 (11.2)	0.1551

PCV13, 13-valent pneumococcal conjugate vaccine; PPV23, 23-valent pneumococcal polysaccharide vaccine.

Multiple regression analysis was used to assess the relationships between several clinical and demographic factors, and likelihood of receiving a COVID vaccine, and both models and odd's ratios were calculated using a GLM (generalized linear model). All variables compared were considered as factors except for age, number of clinic visits and procedures, and years since diagnosis. Two group differences for various factors were compared using Fisher's exact test. All tests were performed using the R “stats” package, version 4.0.4.

## Results

60.3% (813/1349) of IBD patients received at least one dose of either the Pfizer/BioNTech (BNT162b2), Moderna (mRNA-1273), or Johnson & Johnson (JNJ-78436735) vaccines (Table 2). In a multivariate regression, COVID vaccination was found to be positively associated with a number of factors including older age (OR 1.022, *p-*value = 1.65e-5), female sex (OR 1.46, *p* = 0.00194), Asian and White races (OR 2.84, 1.66, *p* = 0.02330, 0.00169), number of clinic visits in the past 12 months (OR 1.37, *p* = 1.11e-08), and biologic use (OR 1.78, *p* = 7.82e-5; Table 3). This was true while controlling for IBD type; marital status; insurance (Commercial vs. Medicaid vs. Medicare); employment status; years since diagnosis; and tobacco, alcohol, and substance use history. Years since diagnosis and age were not found to have a significant interaction suggesting older age independently predicts likelihood of vaccination. There was a negative association between vaccination status and the total number of IBD related surgeries a patient had undergone (OR 0.890, *p* = 0.02857). There was no association between COVID vaccination and the number of endoscopic procedures in the past 12 months, employment status, other types of vaccination (influenza vaccine, 23-valent pneumococcal polysaccharide vaccine, 13-valent pneumococcal conjugate vaccine, recombinant zoster vaccine), or with the use of other IBD medications. 992 patients with IBD received a biologic agent, but only 232, 224, and 233 received steroids, thiopurines or methotrexate, or 5-ASA agents, respectively, suggesting the difference in use may be responsible for the lack of significant relationship between vaccination status and non-biologic treatments. In contrast, age, race, sex, marital status, use of biologic, insurance type, and employment status had no relationship with likelihood of vaccination in those patients without IBD. Only the number of clinic visits a patient had was positively associated with likelihood of receiving a COVID vaccine (OR 1.54, *p* = 0.00383).

**Table 3:** Logistic regression describing predictors of COVID vaccination.

**Variable**	**Odd's Ratio**	**95% Confidence Interval**	***P-*value**
Age	1.02	[1.01,1.03]	1.65e-05
Male gender (relative to female)	0.683	[0.537,0.869]	0.00194
IC (relative to CD)	1.82	[0.896,3.90]	0.109
UC (relative to CD)	0.983	[0.741,1.31]	0.905
Number of IBD related surgeries	0.890	[0.801,0.988]	0.026
Numbers of clinic visits	1.37	[1.23,1.52]	1.11e-08
Numbers of procedures	1.06	[0.901,1.25]	0.500
Years since diagnosis	1.00	[1.00,1.00]	0.406
Positive biologic status	1.78	[1.34,2.38]	7.82e-05
American Indian or Alaskan Native race (relative to Black)	1.17e06	[9.15e-73,NA (undetectable upper bound)]	0.987
Asian race (relative to Black)	2.84	[1.19,7.38]	0.0233
White race (relative to Black)	1.66	[1.21,2.28]	0.00169
Other race (relative to Black)	1.03	[0.463,2.27]	0.951
Married (relative to unmarried)	1.11	[0.846,1.46]	0.446
Commercial insurance	0.545	[0.212,2.47]	0.987
Medicaid insurance	0.269	[0.0877,1.26]	0.275
Medicare insurance	0.413	[0.0744,1.96]	0.987
Employed	6.92e-07	[NA (undetectable lower bound),8.81e+71]	0.988
Unemployed	4.49e-07	[NA (undetectable lower bound), 5.72e+71]	0.987

## Discussion

Our study examined COVID-19 vaccination rates in a diverse, adult IBD and non-IBD population from a single institution in the state of Maryland. 60.3% (813/1349) of our IBD population received the vaccine, which is lower than the 88.4% of the general, adult U.S population and 95% of the Maryland population (as of April 04, 2022) (37). In our study, 67.4% (145/215) of non-IBD patients were vaccinated.

In prior studies of IBD patients, factors such as female gender, younger age, minority race, lack of prior vaccinations, shorter duration of IBD diagnosis, and current steroid therapy, appear to be negative determinants of COVID-19 vaccination in IBD patients (15, 38, 39). Whereas older age (13, 40), male gender (9, 13), White race (13), prior COVID-19 infection (13), prior routine vaccinations (12, 40, 41), current biologics (9, 13), and immunomodulators use (38), and higher education levels (13, 40) were associated with greater incidences of COVID-19 vaccination.

In prior studies examining the relationship between race in IBD patients and vaccination rates, the patient populations investigated were predominantly White. We included a more diverse patient population. Our study also showed that White race was associated with increased vaccination for COVID-19 in IBD patients. But we also demonstrated that Asian race was associated with increased vaccine acceptance, which had not been previously reported. Interestingly, African American race was not a negative determinant of COVID-19 vaccination, as previously demonstrated. However, African American IBD patients were less likely to be vaccinated relative to White and Asian patients. When compared to African American non-IBD patients, African American IBD patients were equally as likely to receive the COVID-19 vaccine. Concentrated efforts must continue to address the many health disparities which have become more accentuated during the pandemic.

Interestingly, in contrast to the other study, our study showed that female IBD patients were more likely to be vaccinated for COVID-19. Our study had 711 (52.7%) female IBD patients and 141 (65.6%) female non-IBD patients. Women in both groups were more likely to be vaccinated against COVID-19 than men. It is possible that non-IBD women were found to be more likely than non-IBD men to be vaccinated for COVID as women comprised a significantly larger portion of the non-IBD population in our study (65.6%).

We found that IBD-related surgeries were negatively associated with COVID vaccination suggesting that patients with history of severe IBD disease may be more hesitant about getting vaccinated. One possible explanation is that the patients fear the vaccine may exacerbate their disease and therefore lead to more traumatic surgeries. Another potential explanation is that patients with more severe disease and more IBD related surgeries may be more likely to be non-compliant with medication (42, 43). Those less compliant with medications are also likely to be less compliant with recommended vaccinations, such as the COVID-19 vaccine.

Interestingly, we did not find an association between prior vaccinations and COVID vaccination in IBD patients as other studies have shown. It is unlikely that IBD patients have an aversion to vaccinations in general as they are significantly more likely than non-IBD patients to receive other types of vaccinations (influenza, pneumococcal, shingles). And, oppositely, non-IBD patients are more likely to be vaccinated for COVID-19 than the routinely recommended vaccines. The IBD patients' reluctance may be due to the relative novelty of the COVID-19 vaccine versus the other vaccinations that have been available for a significantly longer period. Therefore, assumptions should not be made regarding patients' willingness to be vaccinated for COVID based on their vaccination history.

Our study also showed that biologic use was positively associated with COVID-19 vaccine. However, other immunosuppressive medications did not show any relationship as other studies have. This may be because a higher proportion of patients were on biologics (992, 73.5%) at this institution given it is a large tertiary care referring center, and only 232 (17.2%), 224 (16.6%), and 233 (17.3%) received either steroids, thiopurines or methotrexate, or 5-ASA agents, respectively.

We did not find an association between steroid use and COVID-19 vaccination. This may represent a dichotomy of perception of the patients: some may perceive that a COVID-19 vaccination might exacerbate their acute flare, and some may perceive that because they are in an acute flare, they wish to prevent a worse COVID-19 infection outcome.

In congruence with prior studies, older age appears to be associated with more likelihood of being vaccinated for COVID-19 in IBD patients. Age was not a predictor in non-IBD patients. This is likely due to the IBD patients' perception of higher risk for severe outcomes of COVID-19 infection given the evidence that the disease has a higher likelihood of negatively impacting the older population. Another reason why older IBD patients have higher rates of vaccination may be because they have had longer exposure and interaction to the healthcare system and this has required them to have regular contact with their health care providers.

This study also highlights the importance of regular visits with IBD patients, especially those who are immunosuppressed. We demonstrated that both IBD and non-IBD patients with more clinic visits over the 12-month period were more likely to be vaccinated against COVID-19. Clinic visits present opportunities for patients to ask questions regarding the vaccine and how COVID-19 infection can impact their disease. It is possible that patients who have higher numbers of clinic visits represent patients that are in an acute phase of their disease. Therefore, these patients may perceive a heightened risk and vulnerability to COVID-19, leading them to receive vaccinations for COVID-19. In addition, number of clinic visits was the only factor that was positively associated with vaccination in non-IBD patients, which further demonstrates its importance across both populations. Therefore, communication between physician and patient is one of the best contributing factors to getting vaccinated. Unfortunately, it has become easy for patients to be lost to follow-up during the pandemic. As providers, we must continue to educate our patients during clinic or telemedicine visits on the importance of obtaining a COVID-19 vaccine. Often, health maintenance conversations surrounding vaccines can be pushed to the end of the appointment or never spoken about given time restraints. We must continue to make this a priority during appointments given the ongoing global pandemic.

One limitation of this study is relying on the accuracy of the electronic medical record (EMR) for variables including vaccination status; marital status; employment status; and history of tobacco, alcohol, and illicit substance use. Data recorded are based solely on information disclosed by the patient.

In conclusion, greater vaccination efforts should be made for IBD patients, specifically targeting patients that are male, younger in age, African American, and have history of multiple IBD-related surgeries. In addition, efforts should be made to continue regular visits with patients when indicated to improve communication, educational opportunities, and thus increase COVID-19 vaccination uptake.

## Data availability statement

The raw data supporting the conclusions of this article will be made available by the authors, without undue reservation.

## Ethics statement

As this was a retrospective chart review study, individual informed consent was waived and oversight of the protocol was governed by the University of Maryland, Baltimore Human Research Protections Office. All patient data collected were de-identified of any protected health information.

## Author contributions

HK and KP are co-first authors and both planned the study, collected the data, and wrote the manuscript. KP submitted the study. MA performed data analysis and also wrote the manuscript. MBe, EZ, MBo, PP, and LS collected the data. UW was the Principal Investigator who planned and organized the study. All authors contributed to the article and approved the submitted version.

## Funding

MA was supported by T32 DK067872 from the NIDDK.

## Conflict of interest

The authors declare that the research was conducted in the absence of any commercial or financial relationships that could be construed as a potential conflict of interest.

## Publisher's note

All claims expressed in this article are solely those of the authors and do not necessarily represent those of their affiliated organizations, or those of the publisher, the editors and the reviewers. Any product that may be evaluated in this article, or claim that may be made by its manufacturer, is not guaranteed or endorsed by the publisher.
